# Prognostic value analysis of urokinase-type plasminogen activator receptor in oral squamous cell carcinoma: an immunohistochemical study

**DOI:** 10.1186/1471-2407-8-220

**Published:** 2008-08-01

**Authors:** Roberta Bacchiocchi, Corrado Rubini, Elisa Pierpaoli, Giulia Borghetti, Pasquale Procacci, Pier Francesco Nocini, Andrea Santarelli, Romina Rocchetti, Domenico Ciavarella, Lorenzo Lo Muzio, Francesca Fazioli

**Affiliations:** 1 Department of Molecular Pathology and Innovative Therapies, Polytechnic University of the Marche Region, Ancona, Italy; 2 Department of Neurosciences, Institute of Pathologic Anatomy and Histopathology, Polytechnic University of the Marche Region, Ancona, Italy; 3 Section of Oral and Maxillofacial Surgery, University of Verona, Verona, Italy; 4 Institute of Dentistry and Stomatological Sciences, Polytechnic University of the Marche Region, Ancona, Italy; 5 Department of Surgical Sciences, Faculty of Medicine, University of Foggia, Foggia, Italy; 6 Italian National Research Center on Aging (INRCA), Ancona, Italy

## Abstract

**Background:**

Oral squamous cell carcinoma (OSCC) represents the most common oral malignancy. Despite recent advances in therapy, up to 50% of the cases have relapse and/or metastasis. There is therefore a strong need for the identification of new biological markers able to predict the clinical behaviour of these lesions in order to improve quality of life and overall survival. Among tumour progression biomarkers, already known for their involvement in other neoplasia, a crucial role is ascribed to the urokinase-type plasminogen activator receptor (uPAR), which plays a multiple role in extracellular proteolysis, cell migration and tissue remodelling not only as a receptor for the zymogen pro-uPA but also as a component for cell adhesion and as a chemoattractant. The purpose of this study was to gain information on the expression of uPAR in OSCC and to verify whether this molecule can have a role as a prognostic/predictive marker for this neoplasia.

**Methods:**

In a retrospective study, a cohort of 189 OSCC patients was investigated for uPAR expression and its cellular localization by immunohistochemistry. As standard controls, 8 normal oral mucosal tissues free of malignancy, obtained from patients with no evidence or history of oral cavity tumours, were similarly investigated. After grouping for uPAR expression, OSCCs were statistically analyzed for the variables age, gender, histological grading (G), tumour size, recurrence, TNM staging and overall survival rate.

**Results:**

In our immunohistochemical study, 74 cases (39.1%) of OSCC showed a mostly cytoplasmic positivity for uPAR, whereas 115 were negative. uPAR expression correlated with tumour differentiation grade and prognosis: percentage of positive cases was the greatest in G3 (70.4%) and patients positives for uPAR expression had an expectation of life lower than those for uPAR negatives.

**Conclusion:**

The results obtained in this study suggest a role of uPAR as a potential biomarker useful to identify higher risk subgroups of OSCC patients.

## Background

Oral squamous cell carcinoma (OSCC) is one of the most common cancers, with a world incidence of more than 350 000 new cases per year [1]. In spite of improved therapeutic procedures, the mortality rate for this neoplasia has not changed appreciably in the Western world for over 20 years: the 5-year overall survival does not exceed 55% which is mainly caused by locally aggressive tumour phenotypes. At the moment, the most important prognostic factor for OSCC is efficacy of surgical treatment which consists in "complete" excision of the primary lesions and includes intra-operative histopathological examination of peri-tumoral tissues to confirm the presence of tumour-free excision margins. Nevertheless, within 24 months, up to 50% of the cases have relapse and/or metastasis, the possible explanations being inappropriate histopathological evaluation or the presence of "occult" cells that are undetectable by means of the currently used diagnostic procedures. Furthermore, the removal of these tumours frequently needs massive surgical resections that impair important physiological functions such as speech and swallowing. Thus, there is a strong need for the identification of molecular markers that could be correlated with tumour characteristics and able to predict the biological behaviour of an individual lesion in order to differentiate tumours requiring more aggressive therapeutic and/or surgical treatments. This can improve quality of life, disease-free and overall survival of OSCC patients. Even though considerable efforts have been made in this direction, a sensitive and specific marker useful to clinicians to direct preventive and therapeutic management approaches for OSCC has not been identified yet.

Transition from non invasive to invasive carcinoma imply a complex biological change that enables cancer cells to detach from the epithelial cells and to assume the transcriptional program characteristic of mesenchymal cells, in order to acquire the attributes of invasiveness and motility. The most important effectors of these complexes changes are proteolytic systems active on component of the extracellular matrix.

Among them, the proteolytic cascade system of plasminogen activation, directed by the urokinase-type plasminogen activator (uPA) and its receptor (uPAR), has long been recognized as the one that performs a central role in cancer invasion, mainly due to uPAR's capability to concentrate and increase uPA's proteolitic activity on cell surface. In active movement cells, uPAR is localized at the migration front thereby focusing uPA activity where proteolysis is required. Furthermore, uPAR has been recognized as a multifunctional protein connected to a number of non-proteolysis-related processes, such as cell adhesion and migration, chemotaxis and tissue remodelling, emphasizing the importance of this molecule in cell invasion [2].

uPAR expression levels in tumour sections and their prognostic values have been studied in several human neoplasms, such as colon, breast, ovarian, stomach and lung carcinomas [3-5]. These studies have shown that high uPAR expression levels correlate with poor prognosis, therefore making it as a potential biological marker correlated to tumour progression and aggressiveness. Increased uPAR expression has also been found in head and neck squamous cell carcinomas (HNSCC), although without any significant correlation to staging parameters [6]. Instead, an immunohistochemical analysis of 34 primary oral cancers showed a correlation between uPAR expression and both cancer invasion and secondary regional lymph node metastasis [7]. A more recent study of 20 cases of incipient OSCC reported immunohistochemical localization of uPAR in both stromal cells and neoplastic cell islands located at the invasive front of the lesion [8]. This is in line with the emerging concept that morphological and functional characteristics of the invasive tumour front underlie the biological aggressiveness of oral cancer [9]. These results suggest a possible role of uPAR in early oral cancer invasion. Therefore, we sought to extend the analysis of uPAR expression in a larger cohort of OSCC patients in order to gain more information on its relevance for the clinical outcome of these tumours.

## Methods

### Tissue samples and patient characteristics

Histological material was obtained from the files of the Institute of Pathological Anatomy and Histopathology, Polytechnic University of the Marche Region, Ancona, Italy. Samples were taken from oral cavity squamous cell carcinomas and adjacent normal mucosa of 189 patients consecutively recruited between 1990 and 2002. In all cases the specimen provided the first observation of the disease. The patients included 143 males and 46 females. They ranged between 33 and 95 years of age at the time of admission (mean 66.11 ± 0.9 SE); 85 of them had neck nodes and none had any evidence of distant metastasis. Median follow-up was 53.13 months (± 3.4 SE). The diagnosis in this investigation was performed by an expert oral pathologist (C.R.) on the basis of the 2003 World Health Organization (WHO) classification and according to the 2003 revision of the TNM system [10]. Histological grading was done by focusing on the stage of invasion which expresses the infiltrative characteristics of the front of the lesion. On the basis of this classification, the specimens were subdivided into four groups: squamous cell carcinoma grade 1 (G1) (77 cases), grade 2 (G2) (68 cases) and grade 3 (G3) (44 cases), and normal oral mucosal tissues free of malignancy (8 cases) obtained from patients with no evidence or history of oral cavity tumours (Table 1).

**Table 1:** Patient Characteristics

**Group**	**No. of cases**	**2003 WHO classification**	**TNM stage (2003 revision)**			
			**I**	**II**	**III**	**IV**
1	77	G1	41	17	8	11
2	68	G2	28	13	8	19
3	44	G3	10	9	9	16
4	8	normal mucosa				

The use of archived human tissues conformed to an informed protocol that had been reviewed and approved by the institutional review board of the Polytechnic University of the Marche Region, Italy.

### Immunohistochemistry

uPAR expression was evaluated in 4-μm-thick tissue sections cut from formalin-fixed, paraffin-embedded specimens using a labelled streptavidin-biotin kit (Dako Corporation, Carpinteria, California, U.S.A.). One section of each case, stained with haematoxylin-eosin, was used to confirm the histopathological diagnosis. Only those sections that showed sufficient epithelium to assess 500 cells were considered for this study. Antigen retrieval was done by covering the deparaffinized tissue sections with 0.05% Pronase (Dako Corporation, Carpinteria, California, U.S.A.) at room temperature for 10 min in a humidity chamber. After washing in Tris-HCl buffer (0.5 M, pH 7.6), endogenous peroxidase was blocked with 3% hydrogen peroxide in water for 10 min. Next, slides were rinsed in water and Tris-HCl buffer for several minutes before being incubated for 24 h at 4°C with a mixture of two anti-uPAR monoclonal antibodies (R2 and R4, diluted to 1 μg/ml in Tris-HCl, whose specificity in recognising uPAR has been well established [11]). The slides were then washed three times with Tris-HCl buffer and incubated for 30 min with biotinylated goat anti-rabbit and goat anti-mouse immunoglobulins. After extensive washing, slides were incubated with peroxidase labelled streptavidin, subsequently developed using 3,3'-diamonobenzidine tetrahydrochloride (Sigma, St. Louis, Missouri, U.S.A.) and 0.02% hydrogen peroxide. Finally, specimens were counterstained with Mayer's hematoxylin. In all cases, the specificity of the reaction was checked by incubating adjacent sections of each sample with an isotype-matched monoclonal antibody (MOPC-21, Sigma) used at the same final concentration as that of the anti-uPAR R2 and R4.

A mean percentage of positive tumour cells was determined by examining 500 neoplastic cells. Samples were classified as uPAR positive if expression was detected in more than 1% of the cancerous tissue. uPAR- stained sections were also evaluated in terms of cell pattern of staining. This feature refers to the staining of individual cells and it was defined as membranous when uPAR staining revealed predominant membrane distribution, whereas was defined as cytoplasmic when strong positivity was observed in both the cytoplasm and membrane.

### Statistics

Data were analysed using GraphPad Prism software version 5.00 for Windows (GraphPad Software, San Diego California USA, ). Significant differences (*p *< 0.05) between groups were determined using the χ^2 ^– test (Fisher's exact test was used instead of χ^2 ^when only two groups were considered) and Kruskal – Wallis statistic (Mann – Whitney test was used instead of Kruskal – Wallis when only two groups were considered). Survival curves were analyzed according to the Kaplan-Meier method, and for differences between curves the *p *value was calculated by the log-rank test. A *p *value of less than 0.05 was accepted as statistically significant.

## Results

**Figure 1:**
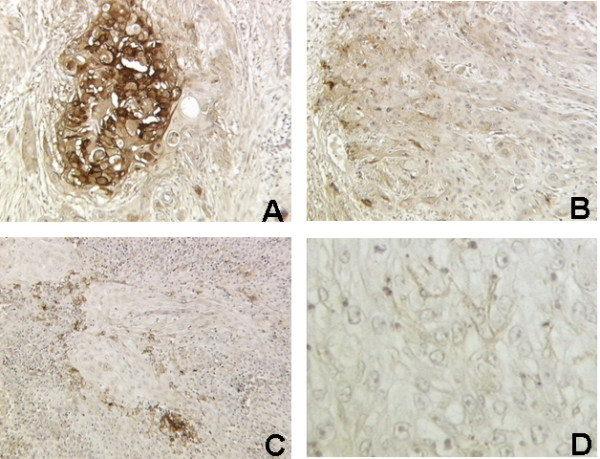
**uPAR expression in OSCC.** A: Strong cytoplasmic positivity of neoplastic epithelial cells in poorly differentiated OSCC (G3) (× 250). B: Weak cytoplasmic positivity of epithelial cells of invasive front in moderately differentiated OSCC (G2) (× 200). C: Focal strong positivity in stromal and inflammatory peritumoral cells in moderately differentiated OSCC (G2) (× 160). D: Negativity of an adjacent section incubated with an isotype-matched monoclonal control antibody (× 400).

Immunohistochemical analysis of cancer tissue samples using specific uPAR antibodies revealed that 74 out of 189 carcinomas were positive (39.1%). The cell pattern of staining, valued in the neoplastic and stromal components of invasive front and inflammatory peritumoral component, was mostly cytoplasmic (Fig. 1). The analysis of 8 normal oral mucosal tissues, free of malignancy, did not reveal any positivity (data not shown).

The obtained data were then used to carry out a statistical analysis, correlating uPAR expression with clinicopathological parameters. The results of this evaluation are shown in Table 2.

**Table 2:** Statistical analysis of uPAR expression and associated clinicopathological findings in OSCCs

**Variables**	**No**.	**UPAr ** **expression** **n. (%)**	**Chi-square ** **P <0.05**	**Mean**	**Standard ** **deviation**	**Standard ** **error**	**Mann ** **Whitney** **P <0.05**	**Multiple Comparison Test**		
Cases	189	74 (39.1)								

Age										
< 65 years	78	25 (32)	No	39	19.8	14	No			
> 65 years	111	49 (44)	0.0989°	55.5	9.192	6.5	0.0854			

Sex										
Male	143	56 (39.1)	No	71.5	21.92	15.5	No			
Female	46	18 (39.1)	1.0000°	23	7.071	5	0.7103			

Grading								**Dunn's Multiple Comparison Test** ** Difference in rank sum P < 0.05**		
G1	77	16 (20.8)	Yes	3.052	8.515	0.9703	Yes	G1 vs G2	-16,14	No
G2	68	27 (39.7)	< 0.0001	5.103	12.09	1.466	< 0.0001*	G1 vs G3	-50,00	Yes
G3	44	31 (70.4)		14.77	20.52	3.094		G2 vs G3	-33,87	Yes

Size										
< 1.5 cm	48	17 (35.4)	No	3.958	8.124	1.173	No			
> 1.5 cm	141	57 (40.4)	0.6092°	7.157	15.28	1.292	0.4607			

Recurrence										
Positive (+)	85	37 (43.5)	No	42.5	7.778	5.5	No			
Negative (-)	104	37 (35.5)	0.296°	52	21.21	15	0.5866			

Staging								**Dunn's Multiple Comparison Test ** **Difference in rank sum P < 0.05**		
I	79	25 (31.6)		4.873	13.47	1.516		I vs II	-10,44	No
II	39	16 (41)	No	6.231	11.45	1.833	No	I vs III	-3,138	No
III	25	9 (36)	0.2307	3.760	7.055	1.411	0.0725*	I vs IV	-22,96	No
IV	46	24 (52)		10.39	17.78	2.621		II vs III	7,302	No
								II vs IV	-12,52	No
								III vs IV	-19,82	No

°Fisher's exact test

* Kruskal-Wallis test

χ^2 ^analysis indicated that uPAR expression statistically correlated with grading. In fact, the number of positive cases was the highest in G3 (31 cases, 70.4%), than in G1 and G2 (20.8% and 39.7% respectively). Moreover, in G3 the percentage of cells expressing uPAR was significantly increased (Table 2).

**Figure 2:**
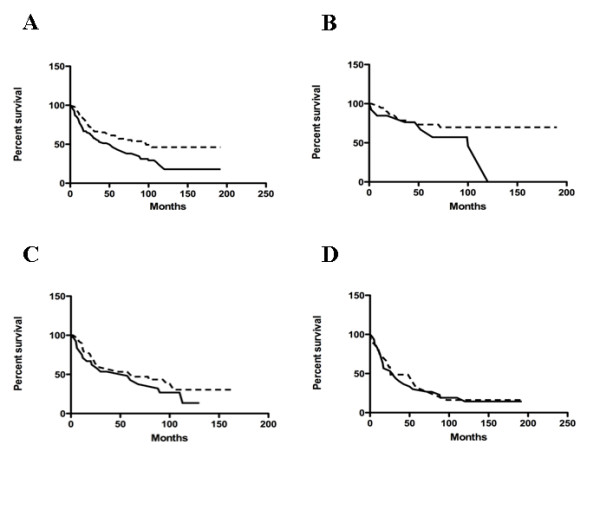
**Prognostic value of uPAR expression.** Kaplan-Meier curves for OSCC patients correlating uPAR expression and overall survival (A) (*p *= 0.0053), survival in grade 1 (B) (*p *= 0.0447), grade 2 (C) (*p *= 0.3427) and grade 3 (D) (*p *= 0.7143) tumours. Dotted line, uPAR negative cases ; continuous line, uPAR positive cases.

To investigate whether uPAR expression was of prognostic relevance, survival analysis was performed and overall survival was calculated using the log-rank statistics. Interestingly, Kaplan-Meier plot, comparing overall survival rates for patients with tumours displaying positive immunohistochemical staining for uPAR versus patients with uPAR negative OSCC (Fig. 2A), revealed a statistically significant association with prognosis (log-rank test, *p *= 0.0053). Patients with uPAR positive tumours had a lower life expectancy than those with uPAR negative tumours. Kaplan-Meier curves correlating uPAR expression with grading showed a statistical correlation between overall survival and grade 1 with a shorter overall survival for uPAR positives patients (log-rank test, *p *= 0.0447) (Fig. 2B), while no correlation was found with grade 2 and 3 (Fig. 2C, D).

Correlation between OSCC that stained positively for uPAR and other variables of potential prognostic significance was studied using the χ^2 ^test. No statistically significant association was observed between uPAR expression and age, gender, tumour size, staging and primary tumour recurrence (Table 2).

## Discussion

Several attempts have been undertaken to improve prognosis in cancer patients and to establish predictive factors related to tumour biology. Presently, for OSCC there are not reliable prognostic factors able to predict the biological behaviour of the tumour or eventual clinical outcome. Clinical stage has some predictive value as does histological grade. However, these parameters suffer from lack of standardization and considerable observed-related variability. There is therefore a strong need for the development of new diagnostic tools that can help the clinician to define the most appropriate management for the individual patient.

Features regarding cells at the invading margins of the tumours are probably of higher prognostic value than features within other parts of the tumours. Several molecular events of importance for tumour spread like gains and losses of adhesion molecules, secretion of proteolytic enzymes, increased cell proliferation and initiation of angiogenesis, occur at the tumour-host interface, where cancer cells receive specific signals from the nearby reactive stroma and undergo an epithelial-mesenchimal transition (EMT). Key effectors of EMT changes are secreted proteases that, by dissolving the surrounding ECM molecules, not only create spaces for invading tumour cells to move, but also mobilize and activate latent growth factors [12]. Therefore, in recent years special attention has been given to tumour cell surface-associated proteolytic enzyme systems such as plasminogen activators and matrix metalloproteinases [13] because of their impact on cancer progression and metastasis [12].

Several kinds of evidence strongly implicate a critical role for uPA-uPAR system in tumour invasion and progression in different types of human cancer [3-5,7,14-20]. In line with these data, our immunohistochemical study revealed a positive correlation between uPAR immunostaining and increasing tumour differentiation grade. However, a comparison of Kaplan-Meier survival curves as a function of grade revealed statistical significance only for G1 patients. A valid explanation for this statistical result is quite difficult based on our incomplete understanding of the molecular mechanisms involved in tumour invasion and metastasis. However, taking into consideration the signalling tumour – stroma interdependence, contribution of molecules, including other protease systems, to cancer aggressiveness can be postulated for cells at various differentiation stages. The total number of proteases made by mammalian cells is vast and we have just begun to analyze the actions of only a small portion of them in the context of cancer pathogenesis. In addition, the expression levels of uPA in oral tumour tissues require a more detailed study.

A recent review indicated that uPA-uPAR can interact with transmembrane proteins to modify multiple signal transduction pathways and influence a wide variety of cellular behaviours [1]. In particular, in pre-malignant oral keratinocytes, matrix-induced integrin clustering results in the activation of Src/MEK/ERK signalling, leading to enhanced uPA expression and re-distribution of uPAR to sites of clustered integrins [21]. Pericellular matrix proteolysis removes physical stimulus for integrin clustering, leading to loss of integrin signalling and subsequent uPA expression [1]. Blocking uPAR-α3β1 integrin interaction using a variety of strategies results in substantially attenuated uPA expression which suggests that uPAR-α3β1 binding potentiates integrin signalling and subsequent pericellular proteolysis [22]. Highly invasive OSCC cells exhibit matrix-independent high-level uPA expression that is not further responsive to integrin clustering [23]. This indicates that loss of adhesion-regulated proteinase expression contributes to OSCC invasive potential [1].

## Conclusion

Our findings reiterate the concept that increased uPAR levels may contribute to the spread and invasion of cancer cells. They also suggest the importance of uPAR as a potential novel prognostic factor in OSCC. This may be potentially relevant for the implementation of closer follow-up protocols and/or alternative therapeutic regimens especially for patients with grade 1 tumours.

## Competing interests

The authors declare that they have no competing interests.

## Authors' contributions

RB participated in the design of the study, performed immunohistochemical study, contributed to the acquisition, analysis and interpretation of data and drafted the manuscript. CR performed immunohistochemical study. EP was involved in drafting the article and in statistical analysis. GB performed selection of archived samples and clinical data collection. PP performed clinical study. PFN performed clinical study. AS was involved in statistical analysis. RR performed immunohistochemical study. DC participated in statistical analysis. LLM conceived the study, participated in its design coordination and performed statistical analysis. FF conceived the study, participates in its design coordination and revised the article critically. All authors read and approved the final manuscript.

## Pre-publication history

The pre-publication history for this paper can be accessed here:


